# Vacuum-Assisted Breast Biopsy After Neoadjuvant Systemic Treatment for Reliable Exclusion of Residual Cancer in Breast Cancer Patients

**DOI:** 10.1245/s10434-021-10847-9

**Published:** 2021-09-28

**Authors:** Vivian Koelbel, André Pfob, Benedikt Schaefgen, Peter Sinn, Manuel Feisst, Michael Golatta, Christina Gomez, Anne Stieber, Paul Bach, Geraldine Rauch, Joerg Heil

**Affiliations:** 1 Breast Unit, Department of Obstetrics and Gynecology, Heidelberg University Hospital, Heidelberg, Germany; 2 Department of Pathology, Heidelberg University, Heidelberg, Germany; 3 Institute of Medical Biometry and Informatics (IMBI), Heidelberg University, Heidelberg, Germany; 4 Institute of Biometry and Clinical Epidemiology, Charité–Universitätsmedizin Berlin, corporate member of Freie Universität Berlin, Humboldt-Universität zu Berlin, Berlin Institute of Health, Berlin, Germany

## Abstract

**Background:**

About 40 % of women with breast cancer achieve a pathologic complete response in the breast after neoadjuvant systemic treatment (NST). To identify these women, vacuum-assisted biopsy (VAB) was evaluated to facilitate risk-adaptive surgery. In confirmatory trials, the rates of missed residual cancer [false-negative rates (FNRs)] were unacceptably high (> 10%). This analysis aimed to improve the ability of VAB to exclude residual cancer in the breast reliably by identifying key characteristics of false-negative cases.

**Methods:**

Uni- and multivariable logistic regressions were performed using data of a prospective multicenter trial (*n* = 398) to identify patient and VAB characteristics associated with false-negative cases (no residual cancer in the VAB but in the surgical specimen). Based on these findings FNR was exploratively re-calculated.

**Results:**

In the multivariable analysis, a false-negative VAB result was significantly associated with accompanying ductal carcinoma in situ (DCIS) in the initial diagnostic biopsy [odds ratio (OR), 3.94; *p* < 0.001], multicentric disease on imaging before NST (OR, 2.74; *p* = 0.066), and age (OR, 1.03; *p* = 0.034). Exclusion of women with DCIS or multicentric disease (*n* = 114) and classication of VABs that did not remove the clip marker as uncertain representative VABs decreased the FNR to 2.9% (3/104).

**Conclusion:**

For patients without accompanying DCIS or multicentric disease, performing a distinct representative VAB (i.e., removing a well-placed clip marker) after NST suggests that VAB might reliably exclude residual cancer in the breast without surgery. This evidence will inform the design of future trials evaluating risk-adaptive surgery for exceptional responders to NST.

Women with breast cancer increasingly receive neoadjuvant systemic treatment (NST).^1^ The use of NST has enabled a better response assessment, more breast-conserving surgeries, and more prognostically favorable pathologic complete responses (pCRs).^2–6^

During the past decade, pCR rates have increased, especially among patients with triple-negative breast cancer (TNBC) and human epidermal growth factor 2 (HER2)-positive breast cancer, with the majority currently achieving ypT0 (pCR-B) status.^7–9^ The increasing pCR-B rates have led to the question whether breast cancer surgery may be omitted for certain patients: For these patients without residual cancer after NST, breast surgery probably is no primary therapeutic procedure but rather a diagnostic procedure without much benefit compared with adjuvant radiotherapy or systemic treatment. However, to date, no other diagnostic procedure except surgery has been able to detect or exclude residual cancer reliably after NST.

In recent years, studies have investigated several approaches to a reliable diagnosis of pCR-B without invasive surgery to allow for risk-adaptive surgery. Imaging (e.g., ultrasound, mammography, positron emission tomography [PET], magnetic resonance imaging [MRI]) showed higher rates of missed residual cancer after NST than after breast surgery.^10–12^ Recently, single-center pilot trials have shown vacuum-assisted biopsy (VAB) to be promising for detecting pCR-B.^13–15^ However, subsequent confirmatory, prospective, multicenter trials could not confirm these findings because the minimally invasive biopsy missed residual cancer more often than expected compared with breast surgery.^16–19^

Decisive guidelines exist for improving the accuracy of VAB in further exploration of the feasibility of omitting breast cancer surgery for women with pCR-B. Factors influencing a false-negative VAB result (i.e., biopsy free of residual tumor but showing residual disease in surgical specimens) are widely unexplored. Also, a consistent definition of the adequate eligible patient cohort and the pathologic and clinical assessment of VAB after NST does not exist to date.

This analysis aimed to improve the ability of VAB after NST to reliably exclude residual cancer in the breast. We used data of the largest prospective multicenter VAB trial (NCT02948764)(18) to identify key characteristics of patients and the VAB procedure associated with a false-negative VAB result. Based on these findings, we then aimed to provide updated patient eligibility criteria and expanded criteria for the use of VAB after NST. This evidence may inform the design of future trials evaluating risk-adaptive surgery for exceptional responders to NST.

## Methods

### Patient cohort

Patients were recruited as part of the prospective, multicenter, diagnostic RESPONDER trial (NCT02948764) evaluating the diagnostic accuracy of VAB to identify or reliably exclude residual disease after NST.^18^ This study was conducted at 21 trial sites in Germany from March 2017 to May 2019. The study enrolled 398 women 18 years of age or older with breast cancer of all tumor biologic subtypes with a partial or complete clinical response to NST.

The clinical/imaging response to NST was evaluated according to national guidelines^20^ by ultrasound and/or mammography and/or MRI as applicable in the clinical routine. The study-specific VAB procedure was performed before guideline-adherent surgery. The guidelines recommended taking at least six biopsy specimens. In this trial, VAB missed residual disease in the surgical specimen after NST for 18 % (37/208) of the patients with residual cancer (false-negative rate).

### Analysis Set

We performed a post hoc exploratory analysis using the full analysis set of the RESPONDER trial (*n* = 398). All the collected co-variables (26 variables) of the original patient cohort were included except for information about Ki-67 due to no established international consensus on data collection of this biomarker.

### Statistical Analysis

Using the full analysis set, we performed descriptive analysis (absolute and relative frequencies) as well as uni- and multivariable logistic regression to identify clinical and pathologic variables (independent variables) associated with a false-negative VAB result (dependent variable; i.e., VAB free from tumor cells but with residual disease in the surgical specimen). All variables with a *p* value lower than 0.1 were included in the multivariable logistical regression using stepwise regression with forward selection. All *p* values lower than 0.05 were considered statistically significant in a descriptive sense (exploratory analyses).

All the statistical tests were two-sided and performed with SPSS Statistics Software version 26.0 (IBM Corp., Armonk, NY, USA). No missing values were imputed.

### Outcomes and Definitions

All the patients underwent VAB (index test) and breast surgery (reference test). Informed by the results of the descriptive and regression analyses, we developed updated exclusion criteria as well as criteria for an uncertain representative VAB. Variables indicating a high risk for a false-negative VAB result that were available before performance of VAB were used to adjust the inclusion and/exclusion criteria for the cohort of eligible patients. Variables available only during or after performance of VAB were used to update criteria for uncertain representative VABs. Per definition of the parental trial, pathologically uncertain representative VABs were defined as biopsies that were unclear or not representative of the former tumor region (i.e., no visible tumor bed).

Next, we applied these updated criteria to the full analysis set to re-calculate the primary end point (false-negative rate) and the secondary end point (specificity, negative predictive values, and positive predictive values) of the RESPONDER trial. The VABs containing residual tumor and uncertain representative VABs were classified as a positive index test. Representative VABs without residual invasive or ductal carcinoma *in situ* (DCIS) cells were classified as a negative index test. A false-negative VAB means that the index test was negative but there was residual disease in the surgical specimen. Consequently, the FNR was calculated as the quotient of negative index tests (VAB) and patients with residual disease in the surgical specimen. The study defined pCR-B as absence of invasive carcinoma and DCIS (ypT0) in the surgical specimen and biopsy material.

## Results

### Baseline and Clinical Characteristics

The baseline demographic and clinical characteristics of the RESPONDER trial are published elsewhere.^18^ Of the 398 enrolled patients, 47.7% (190/398) achieved a pCR. The median age was 52 years (range 24–79 years). After NST, ycT0 status was reached in 43.7 % (*n* = 174), ycT1 status in 48.5 % (*n* = 193), ycT2 status in 6.3 % (*n* = 25), ycT3 status in 0.8 % (*n* = 3), and ycTx in 0.8 % of the 398 patients. All the patients with accompanying DCIS in the initial diagnostic biopsy (not the VAB) (23.1 %, 92/398) had residual invasive disease in the surgical specimen. Ultrasound-guided VAB procedures were used for 78.9 % and stereotactically guided VAB for 20.6 % of the patients.

### Predictors for False-Negative Biopsy

In the performance of the descriptive analysis, the highest false-negative rate was observed for multicentric disease on imaging before (38.5%, 5/13) and after NST (36.4%, 4/11), for patients older than 70 years (28.6%, 6/21), and for accompanying DCIS in the initial diagnostic biopsy (19.5%, 18/92). The lowest false-negative rate was observed for radiographic detection of the clip marker (14.0%, 7/40) or radiographic detection of parts of the lesion (13.4%, 11/82) in the biopsy specimen.

The results of the univariable logistic regression are shown in Table 1. In the multivariable logistic regression, a false-negative VAB result was associated with accompanying DCIS in the initial diagnostic biopsy [odds ratio (OR), 3.94; *p* < 0.001], multicentric disease on imaging before NST (OR, 2.74; *p* = 0.066), and age (OR, 1.03; *p* = 0.034) (Table 2).

**Table 1.** Univariable logistic regression: clinical and pathologic variables associated with a false-negative vacuum-assisted biopsy after neoadjuvant systemic treatment

Variables	OR	*p* Value	FNR (95% CI)
Age	1.03	0.04^a^	
*Tumor biology*			
HER2+	1.65	0.24	25.0 (14.7–37.9)
HR+/HER2–	1.49	0.36	14.1 (7.7–23.0)
TNBC	Reference	–	17.0 (8.1–29.8)
*cT stage before NST*			
cT1	Reference	–	13.7 (7.7–22.0)
cT2	1.37	0.39	20.9 (13.1–30.7)
cT3	2.43	0.15	28.6 (8.4–58.1)
*cT stage after NST*			
ycT0	Reference		22.0 (12.3–34.7)
ycT1	1.13	0.75	13.1 (7.7–20.4)
ycT2	3.72	0.017	27.3 (10.7–50.2)
ycT3	6.19	0.15	50.0 (1.3–98.7)
*No. of biopsies*			
< 6	Reference	–	
≥ 6	1.99	0.16	19.8 (13.9–26.7)
*Categorized as representative by the biopsying physician*			
Yes	2.1	0.13	16.7 (11.6–22.8)
No	Reference	–	30.0 (11.9–54.3)
*Needle size (G)*			
10	Reference	–	14.7 (7.3–25.4)
9	2.56	0.15	30.8 (9.1–61.4)
8	1.45	0.36	21.2 (13.8–30.3)
7	0	0.99	0.0 (0.0–18.5)
*Multicentricity before NST*			
Yes	2.4	0.09^a^	38.5 (15.7–66.0)
No	Reference	–	16.5 (11.8–22.2)
*Multicentricity after NST*			
Yes	3.34	0.05^a^	36.4 (12.8–66.4)
No	Reference	–	16.4 (11.7–22.1)
*Multifocality before* NST			
Yes	0.89	0.84	14.8 (4.8–32.0)
No	Reference	–	18.3 (13.2–24.5)
*Multifocality after NST*			
Yes	1.22	0.76	16.0 (4.4–39.0)
No	Reference	–	17.6 (12.6–23.5)
*Accompanying DCIS in initial diagnostic biopsy*			
Yes	3.65	<0.001^a^	19.5 (12.4–28.6)
No	Reference	–	16.5 (10.6–24.2)
*Radiography: clip marker visible in biopsy specimen*			
Yes	Reference	–	14.0 (6.3–25.7)
No	1.74	0.23	19.6 (12.8–28.2)
*Radiography: lesion of diagnostic biopsy visible in VAB specimen*			
Yes	Reference	–	13.4 (7.3–22.1)
No	1.14	0.75	23.9 (14.8–35.1)
*Microcalcifications before NST*			
Yes	0.88	0.78	12.8 (5.3–24.7)
No	Reference	–	21.2 (15.1–30.2)
*Microcalcifications after NST*			
Yes	0.59	0.41	9.1 (2.4–22.8)
No	Reference	–	21.2 (14.1–30.0)
*Grading*			
G1/G2/Gx	1.51	0.24	17.0 (10.7–25.6)
G3	Reference	–	18.9 (12.3–27.2)
*Tumor subtype*			
No special type	Reference	–	18.5 (13.5–24.5)
ILC	1.06	0.95	10.0 (0.5–40.3)
Other	0.64	0.67	12.5 (0.6–48.0)
Time requirement for VAB	1.01	0.31	
*Clip marker present and within the former lesion*			
Yes	Reference	–	17.2 (12.0–23.4)
No	1.57	0.32	21.9 (10.1–38.5)
*Organizational setting for VAB*			
Ambulant	Reference	–	20.6 (13.4–29.5)
Day of the surgery	2.21	0.79	
In operating room	1.4	0.34	15.5 (8.5–25.0)
Other	1.13	0.13	
*Biopsy guidance procedure*			
US-guided	Reference	–	8.6 (5.7–12.3)
Stereotactic-guided	1.48	0.32	12.2 (6.0–21.3)

*OR* odds ratio, *FNR* false-negative rate, *CI*, confidence interval, *HER2* human epidermal growth factor 2, *HR* hormone receptor; *TNBC* triple negative breast cancer, *NST* neoadjuvant systemic therapy, *DCIS* ductal carcinoma *in situ; ILC* invasive lobular carcinoma, *VAB* vacuum-assisted biopsy, *US* ultrasound

^a^These variables were included in the multivariate logistical regression

**Table 2.** Multivariable logistic regression: predictive factors for false-negative vacuum-assisted biopsy results

Variable	OR (95 % CI)	*p* Value
Age	1.03 (1.00–1.07)	0.034
DCIS^a^	3.95 (1.94–8.04)	<0.001
Multicentric disease before NST	2.74 (0.94–8.03)	0.066
Multicentric disease after NST	1.36 (0.19–9.75)	0.758

*OR* odds ratio, *CI* confidence interval, *DCIS* ductal carcinoma *in situ*, *NST* neoadjuvant systemic therapy

^a^In initial diagnostic biopsy

### False-Negative Rate According to Updated Eligibility and VAB Criteria

**Fig. 1.**
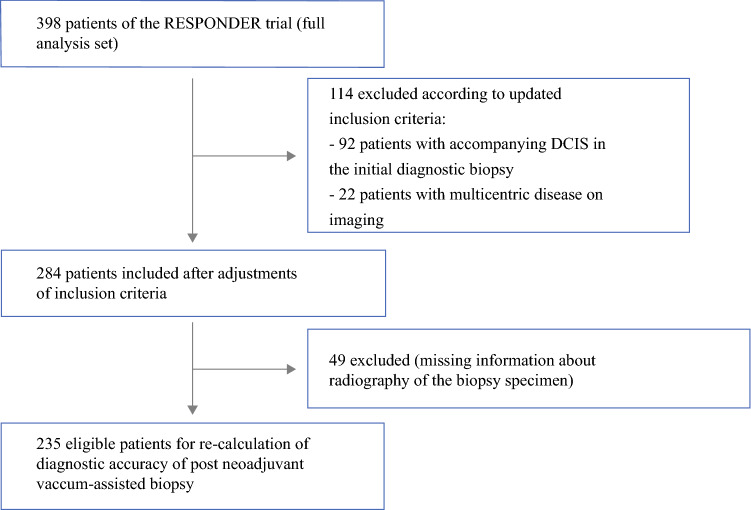
Flow diagram of patients used to calculate the diagnostic accuracy of post-neoadjuvant vacuum-assisted biopsy according to updated inclusion criteria. DCIS, ductal carcinoma in situ

Informed by the results of the uni- and multivariable analyses, we developed updated criteria for eligible patients and the VAB procedure, then re-calculated the false-negative rate. The patients with accompanying DCIS in the initial diagnostic biopsy (not the VAB) or multicentric disease on imaging before NST were excluded (Fig. 1). The VABs that did not remove the clip marker (i.e., clip marker not visible on radiography) and the VABs deemed pathologically to be uncertainly representative of the former tumor region^18^ were considered uncertainly representative.

Table 3 shows the diagnostic performance of VAB after updated eligibility and VAB criteria. The FNR decreased from an initial rate of 17.8 % (37/208) in the primary analysis^18^ to a rate of 2.9% [3/104; 95% confidence interval (CI), 0.1–8.2%]. The NPV increased from 81.4% (162/199) to 93.9% (46/49). The number of eligible patients decreased by 28.6 %, from 398 to 284.

**Table 3.** Diagnostic accuracy of post-neoadjuvant vacuum-assisted biopsy considering updated exclusion criteria and updated criteria for uncertain representative biopsies

	Positive index test: assumed residual tumor	Negative index test: no residual tumor assumed
Residual disease in surgical specimen (*n* = 104)	101	3
No residual disease in surgical specimen (*n* = 131)	85	46
FNR (95 % CI), no.	2.9 % (0.1–8.2), 3 of 104	
Sensitivity (95 % CI), no.	97.1 % (91.8–99.4), 101 of 104	
Specificity (95 % CI), no.	35.1 % (27.0–44.0), 46 of 131	
PPV (95 % CI), no.	54.3 % (46.9–61.6), 101 of 186	
NPV (95 % CI), no.	93.9 % (83.1–98.7), 46 of 49	

*FNR* false-negative rate, *CI* confidence interval, *PPV* positive predictive value, *NPV* negative predictive value

**Fig. 2.**
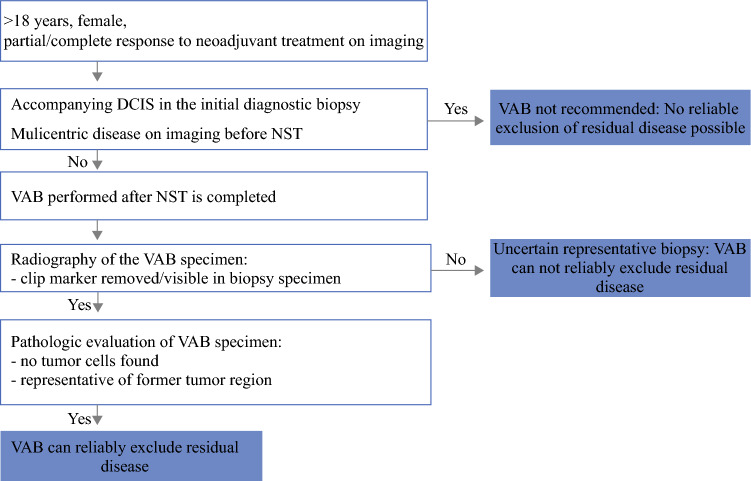
Best practice work flow for the use of vacuum-assisted biopsy after neoadjuvant systemic treatment to reliably rule out residual disease. *DCIS* ductal carcinoma in situ; *NST* neoadjuvant systemic treatment; *VAB* vacuum-assisted biopsy

A best practice workflow for the use of VAB to assess response to NST according to these updated criteria for patient eligibility and the VAB procedure is shown in Fig. 2.

## Discussion

We performed an analysis to improve the ability of VAB after NST to reliably exclude residual cancer in the breast using patient-level data of the largest prospective, multicenter VAB trial.^18^ We identified the key characteristics of the patients and the VAB procedure that were associated with a false-negative VAB result (no tumor in the VAB but residual cancer in the surgical specimen). Based on these results, we provided updated information regarding a possible adequate patient population and the VAB procedure itself.

Patients with accompanying DCIS in the initial diagnostic biopsy or multicentric disease on imaging before NST might not be considered for assessment of response to NST with VAB. Moreover, the analysis might suggest that VABs without removal of the clip marker (no visible clip marker in the biopsy specimen on radiography) should be interpreted as uncertain representative VABs. On the basis of these findings, the FNR decreased to 2.9%. These findings may inform the design of future trials evaluating risk-adaptive surgery for exceptional responders to NST.

The future management of exceptional responders to NST in breast cancer has gained clinical relevance with increasing rates of complete response to NST in recent years. Our results suggest that refinements in the patient selection, VAB procedure, or both are possible and could improve the diagnostic accuracy of VAB.

The question of which patients might be eligible for the omission of breast surgery is under intense debate.^21^ In recent years, mainly four controversial inclusion and exclusion criteria have been repeatedly discussed: invasive cancer accompanied by DCIS, multicentricity, clinical tumor stage, and tumor biology.

In our study, the strongest independent predictors for a false-negative VAB result were invasive cancer accompanied by DCIS in the initial diagnostic biopsy (OR 3.94; *p* = 0.001) and multicentric disease on imaging before NST (OR 2.74; *p* = 0.066). Ductal carcinoma in situ responds differently to neoadjuvant treatment and is associated with higher rates of “scattered” residual tumor after NST. Our study showed that all patients with accompanying DCIS (23.1%, 92/398) in the initial diagnostic biopsy had residual invasive cancer after NST. Previous research showed better pCR rates for patients with accompanying DCIS ranging from 28 to 36%.^22–25^ The lower pCR rates for these patients in our study (0%) might have been attributable to underreporting of patients with accompanying DCIS, and thus a bias toward extensive, non-responding DCIS components may exist. Exclusion of patients with accompanying DCIS in previous trials that evaluated the use of VAB to replace breast surgery could not improve FNR.^26^ Thus, patients with invasive cancer accompanied by DCIS should not be an absolute exclusion criterion for future trials in this area of research. Moreover, the high FNR of patients with multicentric disease on imaging before NST (FNR, 38.5%; OR 2.74; *p* = 0.066) illustrates the relevance of the multicentricity of false-negative VAB results. Patients showing multicentric disease on imaging before NST also might be excluded from future trials.

Another controversial discussion focuses on which tumor stages and tumor biology should be considered for risk-adaptive breast cancer surgery as well as on how to integrate surgical treatment of the axilla. Current research has shown that TNBC or HER2+ breast cancer patients with cT1-2, cN0 status, and a pathologic complete response in the breast (ypT0) have very low rates (< 2%) of residual axillary disease after NST (ypN+).^27, 28^ Thus, these patients should be considered for risk-adaptive breast cancer surgery in future trials. In case of a VAB-confirmed complete response in the breast, they may be spared the operating room completely (omission of breast and axillary surgery).

Advanced age was another finding significantly associated with false-negative VAB findings. Previous studies demonstrated that older patients respond worse to NST, which makes it more likely to miss small, heterogeneous responding tumor foci with a VAB.^29^ However, elderly patients should not generally be excluded from future trials because they may benefit the least from extensive surgery.

Besides refinements in patient selection, our results also suggest further refinements in the assessment of VAB after NST. To date, no established guideline shows which clinical and pathologic criteria should be considered for evaluation of VAB after NST. Although no significant association between the number of biopsy samples and VAB accuracy was observed, we still recommend that at least six biopsies should be taken. Per protocol of the RESPONDER trial,^18^ the pathologic evaluation of the VAB specimen contained an evaluation of the presence of invasive tumor cells and DCIS cells, as well as whether the biopsy material seemed to be representative of the (former) tumor lesion or not.

Although further evaluation of predictive pathologic variables for residual disease such as necrosis or infiltration of lymphocytes may improve the pathologic evaluation, the current assessment could not reliably exclude residual disease (FNR, 17.8%). Our analysis showed that combining the pathologic assessment with the results from specimen radiography of the biopsy specimen to identify the clip marker improved the ability of VAB to reliably exclude residual cancer (FNR decreased from 18 to 3%), but specificity decreased from 85 to 35%, indicating that this condition for the diagnosis of a representative biopsy might be too strong.

For patients whose marker could not be retrieved with VAB, future research may evaluate whether placement of a new clip and its location adjacent to the original clip can improve specificity. Evaluating the importance of retrieving the clip to ensure correct sampling by the physician in a prospective setting is vital. Moreover, future research may evaluate the use of machine learning,^30, 31^ which may allow achievement of a low FNR and a high specificity by identifying complex non-linear data patterns. Previous research on machine learning to improve diagnostic accuracy has shown promising results.^32–34^

When omission of breast cancer surgery is considered, oncologic safety is of utmost importance. The fear of leaving residual disease behind is evident. We should, however, also consider that none of the past de-escalating paradigm shifts in breast cancer surgery have been based on a sensitivity of 100%.^35^ Which FNR is acceptable regarding the detection of residual cancer with VAB after NST needs to be discussed cautiously. With the use of breast-conserving surgery in the early days, higher locoregional recurrences were accepted to implement de-escalation from mastectomy to breast-conserving surgery. As we now know, overall survival was not affected.^36^ Whether and to what extent overall survival would be affected if small residual disease were missed by VAB after NST is unexplored.

Some limitations of our analysis need to be considered. First, this was a post hoc exploratory analysis of a multicenter, prospective trial. Second, although we used the largest prospective trial evaluating VAB after NST, the generalizability of our results to reduce false-negative VAB results cannot be ensured due to the small number of false-negative findings (*n* = 37). Prospective trials to confirm the results of our analysis are indicated. Third, although the current research focused on improving the diagnostic accuracy of VAB to reliably exclude residual tumor after NST, little attention was paid to objective evaluation of our patients’ opinions on options for further de-escalation of breast surgery.^37^ Future trials in this area of research also should address and incorporate our patients’ voice by evaluating our patients’ risk–benefit ratio for future treatment de-escalation protocols.^38^

## Conclusion

For patients without accompanying DCIS or multicentric disease, performing a distinct representative VAB (i.e., removal of a well-placed clip marker) after NST suggests that VAB might reliably exclude residual cancer in the breast without surgery. This evidence will inform the design of future trials evaluating risk-adaptive surgery for exceptional responders to NST.
